# Postscriptum**

**DOI:** 10.1002/bewi.202200028

**Published:** 2022-09-09

**Authors:** Hans‐Jörg Rheinberger

**Affiliations:** ^1^ Max Planck Institute for the History of Science Berlin

Lara Keuck and Kärin Nickelsen, the organizers of this special issue and its workshop, invited me to contribute a closing commentary, and I feel honored and pleased to do so. Now that the English version of the book that inspired it is forthcoming,^1^ it might be better to look ahead instead of looking back. Therefore, I will try to convey in my concluding remarks less the air of a closure than that of an outlook on things to come. And I hope I will be forgiven the rather rhapsodic character of what follows.

I will organize my remarks along the three sections of the issue, Conjunctures, Traces, and Fragments, before concluding with a brief note on historical epistemology. But first, let me comment on the title of my new book: *Spalt und Fuge*, in English, *Split and Splice*. The title was chosen with deliberation. *Spalten*, to split, and *fügen*, to splice, are the two cardinal activities of experimentation. I consciously avoid the traditional notions of analysis and of synthesis. They are logical categories that have been imported into the practice of experimentation; they have not grown out of it, and they suggest neat divisions and equally neat fusions. Neither is characteristic of the experiment. Experimentation, as a process of finding one's way into the unknown, needs more practice‐oriented categories in order to apprehend its moves. If you split a log, the wood resists, and the products of your wedging activity will show uneven faces, depending on the knots and inner structure of the trunk. The same holds true for the object of your experimental inquiry; knowledge of these structures is of utmost importance for experimental exploration. If you splice a rope or if you graft a twig onto your vine, the points of suture will remain visible as signs of a mutilation. So will the pieces of your experimental activity, if joined to form a whole again. And it is indeed of utmost epistemic importance for the ongoing experimental process not to forget that these sutures always are—and will have to be—provisional. The title of this phenomenology of experimentation, *Split and Splice*, aims at calling to mind these epistemic uncertainties, inherent in the life of epistemic things.

## 1. Conjunctures

In scientific investigation, the most minute procedures are of the highest importance. The lucky choice of an animal, an instrument construed in a specific manner, the use of one reagent instead of another, will often suffice to resolve general questions of a highest order. […] In one word, the greatest scientific truths have their roots in the details of experimental investigation that constitute in some sort the soil in which these truths develop.^2^

With these words, taken from his *Introduction à la médecine expérimentale*, Claude Bernard captures the deep roots of contingency in experimentation. Conjunctures, the crossing points of independent chains of events, are conditioned by such contingencies. They are the forms in which the moment of the aleatoric plays out in scientific development, in which the unforeseen materializes. Conjunctures are at the heart of scientific experimentation, and they happen at the level of experimental systems as well as at the level of experimental cultures. A conjuncture, in our context, means an event that is usually triggered by minutiae, but one that reveals itself to have major consequences. The life‐worlds of experimentation are replete with such events. Gaston Bachelard addressed these life‐worlds as “cultures” that give “access to an emergence.”^3^ In autobiographical reminiscences of the actors, they often take the narrative form of having been in the right place at the right time. It is not by chance that this phrase highlights the experimental circumstances with their peculiar affordances at the expense of the action radius of the players. In the sociological literature, taking its starting point from the early and late writings of Robert K. Merton, the term “serendipity” has become common currency as a notion apt to capture the eventfulness of the research process.^4^

Conjunctures occur in many guises and sizes. At the level of experimental systems, they can concern the technical conditions of the research process, that is, the combination and recombination of research technologies. I addressed these occurrences as activities of grafting in a chapter of *Spalt und Fuge*,^5^ a term that derives from the arsenal of biological techniques of cultivation. Conjunctures can also concern the interface between instruments and the targets of research, the epistemic things. In the life sciences, the intricacies of these interfaces—friction surfaces between bits and pieces of biological material and physical or chemical instrumentation—have always been and will continue to be major *loci* of resistance and surprise in the research process. At the level of experimental cultures, conjunctures can take the form of a proliferation or a fusion of experimental systems. And at the level of disciplines, the twentieth‐century life sciences were characterized by major events of hybridization—of physics and biology to biophysics; of chemistry and biology to biochemistry; of biophysics, biochemistry, and genetics to molecular biology, to name only the most prominent of these conjunctures. For a historical epistemology that aims at thinking about history less in terms of persons, ideas, and influences, and more in terms of structures, series, and transformations,^6^ conjunctures are an essential category to come to terms with the intricacies of a non‐deterministic process in which genuine novelties can arise and take shape.

In one way or another, all the case studies collected in this section of the special issue deal with conjunctures in the history of twentieth century life sciences, *glückliche Fügungen*, as the title of one of the contributions aptly says. And, finally, why shouldn't we look at a workshop, such as the one whose results are presented here, as a place of conjunctures? The confluence of independent, but not unconnected research trajectories always gives rise to flashes of new insight.

## 2. Traces

In *Spalt und Fuge*, I distinguish between the experimental space of traces and the space of data, that is, between the order of the graphematic and the order of representation. Not least, this may help to situate what we call data in today's age of big data. Traces are the direct products, the primary outcome of an experiment. They originate at the interface between the epistemic target and the apparatus used to investigate that target. Traces, volatile as they usually are in character, have to be transformed, made durable, and stored in one way or another in order to serve as data and be manipulated in the data space, freed from the temporal constraints of the experiment. In this process of translocation, the traces undergo a change from the medium of the experiment to a medium of a different grain and materiality, be it wire, paper, or the digital. The figurations that we are used to calling models in experimental science only originate in the space of data. But models are not the sole figurations that become possible in the data space. Data can be made to undergo other transformations, such as listing, filtering, ordering according to variable criteria, storing, curating, you name the options. The data space is much more malleable than the space of traces, and working in the space of data does not meet with the kind of resistances that the materiality of the experimental process imposes on the experimenter. This makes it all the more necessary to explore its own intricacies.

Due to electronic data processing, the data space has undergone an unprecedented growth in the past half century. Not that it did not exist before: the conventional world of protocols is a data space, but its immense growth has unleashed a dynamic that lets it appear as a genuine space of its own. What this means for the professed traditional way of doing experiments in relation to the data space and what it means to transform the data space itself into an additional space of experimentation has only started to be thoroughly explored.^7^ A number of papers in the present issue touch on questions related to this problem. The data space in its own materiality and with the inherent recalcitrance of its own practices deserves, and is in need of, the same attention that scientific practice has experienced since the practice turn in the history, philosophy, and social studies of science. Otherwise our studies remain in the realm of the proclamations of its various stakeholders, and with that, to use Georges Canguilhem's expression, in the realm of “scientific ideology.”^8^ The traditional resources for studying science in practice have been recruiting laboratory experience, doing field work, and exploiting archival sources, in short, looking into what François Jacob called the “night” side of the sciences.^9^ All three of these resources are necessary to thoroughly studying the data space as well. And as understanding the language of the sciences is a prerequisite for the study of scientific practice, so is understanding the language of the algorithms, or software, for the study of data practice.^10^ For a critical engagement with data science, its black boxes need to be opened just as the black boxes of instrumentation and of the experimental sciences had to be cracked in the transition from publication‐oriented to practice‐oriented studies of science.

## 3. Fragments

*Spalt und Fuge* closes with a chapter that I have called a “eulogy” of the fragment.^11^ Traditional epistemic wisdom looks at the fragmentary as a deficient state of things, eventually to be surmounted by a knowledge of the whole, just as it looks at ignorance as a state of the mind eventually to be overcome by enlightened knowledge. Here, I argue in contrast that we should look at the fragmentary, just as we should look at ignorance, as driving forces of the research process and thus try to understand their positive qualities. Fragmentation is an activity as well as a state of being that characterizes both the natural sciences and the historical humanities. But there is also a difference between the two realms of knowledge acquisition. While the sciences have to break up their materials in order to gain knowledge about their fine structure as well as the relations between and the functions of their parts, the work of fragmentation has been carried out for the historical humanists by time itself, by the inevitable cassations of the archive. They find their materials already in a fragmented state, and their work is essentially a work of reconstruction from more or less accessible leftovers. And actually, the same holds for the biological and the physical sciences of the past, such as paleontology, earth history, and astrophysics.

One is reminded here of the hyperbolic claim of Georges Cuvier, the French founding figure of paleobiology, that it is possible to derive the shape of the animal as a whole from one of its bones or teeth, actually a belief built on the holistic intuition that all parts of a living being stand in correlation to one another.^12^ We know of a form of historical narration that follows a similar structure: the case study. A case study, in contrast to mere local history, aims at having the particular case stand in for any other given case in a broader historical ensemble with the assumption that they are all interconnected. Case studies are fragments, accessible, and telling, metonymic as they are, yet they can convey their message in a more powerful way than a lengthy juxtaposition of ever more surrounding and filling details could ever do.

Parsimony is a more powerful epistemic tool than any aspiration to completion and totality could be. Fragmentation is not only a necessity in the realm of knowledge acquisition, it is its driving force, and it is a permanent challenge. We encounter it in the innermost process of experimentation as well as in the stories we tell ourselves about the history of the sciences. Whether we look at the traces that are being generated in an experiment or at the models we derive from them, whether we look at the delineations of what Bachelard called the “cantons” of the “scientific city”^13^ or at our own ways, as historians, of dealing with these demarcations, the epistemic challenge as well as power does not come with filling in, but with leaving out. Epistemic intervention as well as representation, to pick up on Ian Hacking's distinction,^14^ are both built on the fragmentary, on omission. Gaps are not a shortcoming, on the contrary, they are constitutive of the mode of engaging with the world that we can call epistemicity. Our mappings of the world rely on intelligent parsimony.^15^ A map aspiring to be coextensive with the mapped would make no sense anymore, its supposed perfection would amount to an absurdity, as has frequently been remarked.^16^ The notion of fragment stands in here for a whole field of related states of things and activities. What unites them is their resistance to plenitude: gaps, leftovers, omissions, and not least, the trace itself as a particular kind of fragment pointing to the absence of something that nevertheless left its imprint, as its absent presence, at the point of contact as an absent origin.

## 4. Historical Epistemology: Then and Now

Historical epistemology is not a doctrine, it is an ongoing process. It is for this reason that I prefer to speak of historicizing epistemology instead of historical epistemology *tout court*.^17^ This process started on the margins of late nineteenth century positivism, and it has grown in waves over the twentieth century: the interwar period, the 1960s, and the turn of the twenty‐first century.^18^ The historicization of epistemology has assumed, over time, different shapes, and it will continue to do so, not least because its subject, the historical development of the sciences, is permanently assuming new and different shapes. An epistemology that strives to live up with these developments must itself be ready to permanently question its orthodoxies, to be constantly on trial.

Therefore, we should become acquainted with the idea of thinking about the historicization of epistemology itself as an empirical, ongoing, experimental endeavor. Since nobody can tell and know in advance which of the experimental lines pursued will be successful in the long run, be it in the sciences or in the historical discourse about them, the field is open to explore different avenues and takes. Whether the various approaches will cancel each other out or whether they will enter into resonance with each other and broaden the field will only find its answer in the ongoing process itself. Scientific rationality is not a timeless mind‐frame. It evolves and changes in and with time. Reflecting about and understanding the historicity of scientific rationality is bound to be subject to comparable evolution and change as well. This is actually not a completely new insight. Gaston Bachelard already has formulated it succinctly in his *Le rationalisme appliqué*: “Epistemology has thus to be as mobile as is science.”^19^ As long as there is a future for the sciences, there will be a future for historical epistemology.

## Footnotes

n1 Rheinberger 2021; Rheinberger, in press.

n2 Bernard 1984, on 44.

n3 Bachelard 1949, on 133.

n4 Merton 1968; Merton and Barber 2006.

n5 Rheinberger 2021, Chapter 4.

n6 Foucault 1972.

n7 See, e. g.: Gramelsberger 2011; Leonelli 2016; Varenne 2019.

n8 Canguilhem 1977.

n9 Jacob 1998, on 126.

n10 Fischer et al. 2020.

n11 Rheinberger 2021, Chapter 10.

n12 Cuvier 1818.

n13 Bachelard 1949, on 132–133.

n14 Hacking 1983.

n15 On what we might call a philosophy of mapping, see Toulmin 1953.

n16 Most famously in Borges 1935.

n17 Rheinberger 2010.

n18 Rheinberger 2022.

n19 Bachelard 1949, on 10.
